# Health care payment practice, perception and awareness of national health insurance scheme by market women in Enugu Metropolis South-East Nigeria

**DOI:** 10.11604/pamj.2021.40.127.26775

**Published:** 2021-11-02

**Authors:** Ebelechukwu Lawretta Okiche, Chikosolu Yvonne Okiche, Chima Theresa Isife, Clara Chinasa Obi-Ochiabutor, Chukwunweike Anukenyi Ogbuabor

**Affiliations:** 1 Faculty of Law, University of Nigeria, Enugu Campus, Enugu State, Enugu, Nigeria,; 2 Department of Paediatrics, Alex Ekwueme Federal University Teaching Hospital, Abakaliki, Ebonyi State, Ebonyi, Nigeria,; 3 Institute for Development Studies, University of Nigeria, Enugu Campus, Enugu State, Enugu, Nigeria,; 4 Faculty of Law, University of Nigeria, Enugu Campus, Enugu State, Enugu, Nigeria,; 5 Ministry of Justice, Enugu State, Enugu, Nigeria

**Keywords:** Healthcare payment, awareness, national health insurance scheme, informal sector, market women

## Abstract

**Introduction:**

health insurance is in the fore front of health financing and achievement of universal health coverage for all. It provides a means of coping with some of the risks faced by individuals in achieving optimal healthcare. Women are vital in the family especially when it comes to the health of their children. We therefore set out in this study to assess the healthcare payment method of women in the informal sector and their awareness of the National health insurance scheme (NHIS) in Nigeria.

**Methods:**

the study was a cross sectional descriptive survey involving women traders who were aged 18 years and above. Three hundred and fifty-three (353) womens were recruited using semi-structured interviewer questionnaire and data was analyzed using SPSS version 22.

**Results:**

the prevalence of awareness of NHIS among the women was 57.5% and educational status was contributor to awareness of the scheme. Also, only 9.9% of the women were registered under the NHIS and out of pocket payment for healthcare was practiced by as much as 88.7% of the respondents. Most respondents had poor perception about the scheme with 60% believing that the government cannot be trusted to keep its end of the bargain with regards to the NHIS.

**Conclusion:**

the need for awareness of the NHIS especially in the informal sector for women is brought to fore. The policy -makers should take into account how women in informal sectors should be captured to enrol in the NHIS in order to expand its coverage and this enrolment should be compulsory.

## Introduction

The most important of all human rights is the right to life for without it other rights accruing to human beings become useless. On the other hand, for life to be meaningful, it must be healthy. In developing countries like Nigeria, access to healthcare is impeded by a lot of factors but top on the list is financial capacity [1]. Healthcare seems to be reserved for only the well to do because out of pocket payment for healthcare is the order of the day [1]. Payment for healthcare can be either by pre-payment (insurance) or out of pocket payment (OPP). OPP for healthcare is fraught with a lot of problems such as late presentation to hospitals, using substandard health services or even not seeking healthcare when sick due to lack of money [2]. This can adversely affect the country´s health indices all round. For example, Nigeria accounts for 10% of global maternal mortality with 59,000 women dying annually from pregnancy and child birth; only 39% are delivered by skilled health professionals [3]. One major reason for this happening is lack of financial capacity.

The provision of robust health services for the preservation and enhancement of life has always agitated the mind of every responsive government the world over. No wonder the whole world, through the United Nations (UN) Sustainable Development Goals (SDG) seeks to “ensure healthy lives and promote well-being for all at all ages,” (SDG3) [4]. On its part, the Nigerian Constitution provides for the right to adequate medical and health facilities for all persons [5]. To give effect to this, the government has continually enacted laws and policies some of which include the National Health Act (NHA) 2014 [6], revised National Health Policy (NHP) 2016 [7] and National Health Insurance Scheme (NHIS) Act 1999 [8]. The question here is, ‘Is this enough? Health insurance eliminates the problem of direct financing or OPP for medical care when a person needs it. However, health insurance is a relatively new concept in the many African countries, including Nigeria [9]. It has been found to be by far the best way of achieving universal health coverage. According to the World Health Organization (WHO), continued reliance on direct payments, including user fees, is by far the greatest obstacle to progress in achieving universal health coverage [10]. The answer is to move towards a system of prepayment and pooling, sharing the financial risks of ill health across the largest population group possible and this in effect means “that the rich subsidise the poor, and the healthy subsidise the sick´ [10].

In order to provide equitable distribution of health, the National Health Insurance (NHIS) was introduced in Nigeria. The scheme was launched in Nigerian on October 15, 1997 and was passed into law in May 1999. It has under gone various amendments since then. The scheme´s main objective is to provide affordable healthcare services to insured individuals and their dependent thereby providing easy access to healthcare towards achieving universal health coverage for all [11]. The national health insurance scheme is still a challenge in the informal sector and rural areas in Nigeria because health insurance is yet to be made compulsory for all citizens. Under the NHIS, informal sector workers are expected to enroll voluntarily by making annual premium payments. The benefits of implementing the Compulsory Health Insurance Scheme include three aspects: it provides for the pooling of resources for subsidisation of health costs where those in high-income bracket support those in low income bracket; the healthy subsidise the sick and the young subsidise the old; the burden of funding healthcare services is shared between government and private employers and enrollees [11].

The aim for instituting NHIS is yet to be achieved as there is still very low coverage. Sadly, less than 10% of Nigerians are enrolled in the NHIS; more so those enrolled are people in the formal sector who are employed and can most times afford to pay for healthcare services [11]. The low coverage is probably because of the challenges facing the scheme. These challenges include inadequate legal framework for a successful scheme, poor implementation of the Act, poor government funding of health care and the health insurance scheme, optional enrolment policy, inappropriate practices by the regulatory agency, the health maintenance organizations and the providers as well as poor political will [11]. These challenges impede the uptake of the scheme by the citizen. Studies [12,13] from different parts of the country have shown that knowledge about the insurance schemes especially among the informal sector is low. The informal sector comprises mostly of the poor and vulnerable groups who are unable to pay for healthcare or work in places where it is difficult to collect insurance contributions [14]. Whereas among the formal sector, studies [15,16] have shown that knowledge is better though enrollment is still not optimal despite willingness to enroll.

Most studies about NHIS have been done in urban communities, the formal sector and among men and women but we thought to work with women who are in the informal sector. Women were chosen because a lot depends on them in the dynamics of the family. They are the most vulnerable and what happens to children health wise in the family depends largely on the woman. Aregbesola and Khan [17] in their survey discovered that as much as 97.9% of women of reproductive age in Nigeria were not covered by health insurance. The aim of this study is to assess the healthcare payment practice, awareness and perception of the NHIS by market women in Enugu Metropolis Southeast Nigeria and find out factors associated with these practices.

## Methods

**Study area**: Nigeria is administratively divided into states, with each state made up of varying number of Local Government Areas. Enugu is the capital city of Enugu State, with a population of about 3 million people. This study was carried out in Enugu Metropolis which has 3 local government areas but Enugu North and South Local Government Areas are chosen. The study was carried out in Ogbete, Mayor and Kenyetta markets in Enugu. These were chosen because they were the biggest markets in the city which had more women.

**Study population**: the study was among women traders in the markets chosen. The majority of the women traded in goods which includes household items, clothing, foodstuffs etc. Only market women, who were adults 18 years and above, who owned stalls i.e. physical location in the market, were studied.

**Study design**: the study design was a cross-sectional survey.

**Sample size**: sample size was estimated using the result of a previous study where the proportion of those who had a good knowledge of the NHIS was 28.7%, [12] with a power of 80% and a confidence level of 95%. This yields a sample size of 335, and adjusting for a 5% non-response rate, a total of 353 respondents were estimated for the study. Using the formula for allocation of proportion, having found out the total number of women in each market visited, the number of women to be sampled in each market was got.

**Ethical approval**: Ethical approval was got from the Ethics Review Board of the University of Nigeria Teaching Hospital, Enugu, Nigeria. Certificate reference number: NHRE/05/01/2008B-FWA00002458-1RB00002323. Also informed consent was got from all the study participants before data collection.

**Sampling technique**a multistage sampling technique was used in the choice of respondents for the study as follows: Using purposive sampling technique the three markets in the Enugu Metropolis were selected. This was based on markets where more women will be found. The number of market women in each market was got from the union leaders. For selection of stalls, systematic sampling was used. Every fifth stall was selected in each market visited and when the owner of the stall is not female, the next fifth stall was chosen till the sample size was completed.

**Data collection**: a semi-structured, pretested interviewer-administered questionnaire was used to collect the data. Data collection was done over a 3 month period (May 2020-July 2020). The questionnaire was developed based on the study objectives and adapted from a previous similar study [12, 18]. The questionnaire was translated to local language for those who are unable to understand English. A pre-test field exercise of the questionnaire was carried out in another market close to the study area but within the same LGA. Verbal informed consent was obtained from all the participants before interview and the questionnaire was administered to one stall owner or supervisor per stall.

**Data analysis**: the data was analysed using SPSS version 22. Frequency tables were generated. Chi-square test was used for categorical data to test for associations. Level of statistical significance was set at p < 0.05.

## Results

**Sociodemographics**: a total of 353 women were recruited from the 3 markets visited. The age of the respondents was between 18 years to 70 years with the mean age being 39±5. Majority of the respondents were married (58.1%) and most of them had some form of formal education. Also 98.8% of the respondents were Christians as shown on Table 1.

**Table 1:** sociodemographic characteristics of participants

Characteristics (N = 353)	Frequency	Percentage (%)
**Age (years)**		
<40	192	54.4
≥ 40	161	45.6
Mean age	39.0± 5	
**Marital status**		
Married	205	58.1
Others*	148	41.9
**Religion**		
Christianity	349	98.8
Islam	4	1.2
**Educational status**		
No formal education	22	6.2
Primary/secondary	229	64.9
Post-secondary	102	28.9

* Others= single, married and widowed

**Knowledge of NHIS**: the prevalence of awareness of NHIS among the women was 57.5%. Most of the women got the information from family and friends (34.3%) and Television (32.8%). Also, a good number of the participants (41.9%) did not know that private individuals and people working in the informal sector could register under the scheme. Table 2 shows that the key contributors to awareness of NHIS are education and religion as women who had post primary education or more were more likely to be aware of NHIS (X^2^= 15.246, p 0.000), so also Christian women (X^2^= 5.475, p 0.019).

**Table 2:** sociodemographic factors and relationship with knowledge of NHIS among the participants

Determinants	Knowledge		Total (%)	Chi-Square	p-Value
	No (%)	Yes (%)			
**Age (years)**					
≤ 40	80(41.7)	112 (58.3)	192 (100.0)	0.118	0.732
>40	70 (43.5)	91 (56.5)	161 (100.0)		
Total	150 (42.5)	203 (57.5)	353 (100.0)		
**Marital Status**					
Others	68(45.9)	80 (54.1)	148(100.0)	1.243	0.265
Married	82 (40.0)	123 (60.0)	205 (100.0)		
Total	150 (42.5)	203 (57.5)	353 (100.0)		
**Religion**					
Muslim	4 (100.0)	0 (0.0)	4(100.0)	5.475	0.019
Christian	146 (41.8)	203 (58.2)	349 (100.0)		
Total	150 (42.5)	203 (57.5)	353 (100.0)		
**Highest Level of Education**					
Primary or No Education	36 (66.7)	18(33.3)	54 (100.0)	15.246	0.000
Post-Primary	114 (38.1)	185 (61.9)	299 (100.0)		
Total	150 (42.5)	203 (57.5)	353 (100.0)		

**Perception of the Women Traders about of the NHIS**: on perception, the respondents were expected to answer Yes or No to the questions and Table 3 represents the perception of the study participants about NHIS. More than 80 % of the respondents perceived NHIS as that which will reduce their out-of-pocket expenditure on health. Also 73.4% of the respondents are willing to register in the NHIS scheme. However, only 50.1% of the respondents think they can trust the government to deliver its promises on the NHIS.

**Table 3:** perception of the women traders about NHIS

Perception	Frequency	Percent (%)
Thinks the health insurance scheme in Nigeria is trustworthy	192	54.4
Thinks the government can be trusted about NHIS	177	50.1
Willing to register under NHIS	259	73.4
Thinks a health insurance scheme is necessary	296	83.9
Thinks NHIS will reduce your out-of-pocket expenditure on health	296	83.9

**Payment practices of women traders in obtaining healthcare services**: on the payment practices, only about 13% of the respondents were registered under any form of health insurance, hence majority of them (88.7%) practiced out of pocket payment for health care. This also reflected in where the women sought for healthcare service as about 72% of them said that the amount of money during an illness determines where healthcare service will be sought for. Also only about 18.7% of the women set money aside regularly for health purposes. Table 4 represents the practices of the participants concerning healthcare payment and NHIS.

**Table 4:** healthcare payment practices of the study participants

Payment practices (n = 353)	Frequency	Percent (%)
Registered under any form of health insurance	46	13.0
Regularly set money aside for health emergencies	66	18.7
Mode of payment	313	88.7
Out of pocket	313	88.7
Health insurance	35	9.9
Borrowed to pay for medical bill	183	51.6
Get support from friends and relatives to pay hospital bills	254	72.0
Sell property to get money to pay hospital bill	58	16.4
Amount of money determines where healthcare is sought for	254	72.0

## Discussion

The study participants were mainly young and middle aged women with mean age of 39years ±5. Knowledge of the insurance scheme was low as only 57.6% of the women have heard about NHIS. This result is comparable with that found in the study by Abdulkaheem *et al*. [13] in contrast, among artisans in Osun State the deficiency in knowledge of NHIS was higher [12]. Reason for this may be because of the varied population that was studied in both studies. Our study was among women alone in the informal sector.

Knowledge was higher among women who had post primary education or higher. There was also a significant relationship statistically between knowledge of NHIS and level of education with level of education being a contributor to the awareness. Better educated individuals are usually more able to access diverse sources of information, process it adequately and take advantage of inherent benefits therein than those who are less educated and those without formal education. This is in keeping with other studies [17,19,20] which found that level of education is a predictor of knowledge of NHIS and subsequent enrollment. 80% of the respondents reported to paying out of pocket for health care. This is consistent with findings in other developing countries where pre-payment for health care is yet to become the norm [21]. This method of payment predisposes to late presentation, limited health care or failure to seek care at all. This affects our country´s health indices adversely. Recently Nigeria was named one of the countries contributing the highest to world infant mortality. This is not surprising. In our study, most of the women agreed that the amount of money they had will determine where healthcare will be sort for. It is no surprise then that these women with their children when sick may patronise traditional unorthodox medicine and other substandard health care as these are cheaper options. The cycle of more morbidity and mortality therefore continues. It´s known that people are most likely to seek good health care and on time if funds were not a problem.

Furthermore, in our study we found out that only about one-tenth of the women were registered under any form of health insurance with only about 17% of them regularly putting money away for health emergencies. The fallout from this is that they borrowed to pay for health services or depended on support from friends and family for healthcare. These reported strategies are evidence of financial insufficiency and difficulties such that can impede access to good health care services. There is need, as a matter of urgency, for health insurance to reduce financial hardship and in turn improve access to health care services. With the agitation for universal health coverage for all by the WHO, pre-payment and health insurance is the only way this can be achieved. Nigeria is already taking a step in the right direction by the NHIS. However a lot still needs to be done. Again, from our study, the perception of the women on the NHIS was good, majority of them were willing to register under the scheme as they believed that the scheme will help reduce OPP for healthcare. This perception is similar to findings in other studies which have shown that potential beneficiaries and health policy makers were interested in health insurance and expressed their willingness to participate [13, 15]. However, the level of trust in the scheme was low. Most of the women did not believe that the government could be trusted to keep their own end of the bargain in the scheme. The main concerns were reports of embezzlement of funds meant for the scheme and lack of transparency, corruption, cultural and religious beliefs. Odeyemi and colleagues [22] in their study also had similar findings among their participants that low level of trust in government polices was an impediment for people enrolling in health insurance scheme. This brings to fore the need for a reform in the insurance scheme whereby only trusted individuals with integrity will be put in charge of the funds.

## Conclusion

In our study, we found out that women in the informal sector of the area studied had poor knowledge of the NHIS and this translated to the fact that enrollment into the scheme by the participants is very poor. We therefore recommend the following: 1) an amendment of the enabling law to make the informal sector enrollment compulsory. The case of Rwanda is very instructive here. Prior to the year 2008 health insurance was voluntary in Rwanda and the scheme suffered from low enrollment just like in every other country in Africa; the moment it became compulsory, enrollment increased by leaps and bound. By 2010, 92% of Rwandans (the highest in Africa) participate in the Scheme [23]. 2) Aggressive public enlightenment and awareness creation especially at the grass root through various channels such as churches, market, village meetings and trade and professional associations. 3) The government needs to up its art by showing enough political will to make the scheme work by a strong regulatory oversight to not only checkmate corruption but also to engender trust and eschew skepticism with which Nigerians view government projects.

### What is known about this topic

- There is poor coverage of the NHIS in Nigeria especially among people in the informal sector;
- Out of pocket payment for healthcare is still very rampant in Nigeria.

### What this study adds

- The very low level of participation in the NHIS specifically among women in the informal sector;
- Poor trust in the NHIS which affects enrolment into the scheme;
- The poor practice of regularly putting money away for health emergencies by the women despite majorly practising out of pocket payment for healthcare.
